# Usage of the H3 variants during the S-phase of the cell cycle in *Physarum polycephalum*

**DOI:** 10.1093/nar/gkac060

**Published:** 2022-02-07

**Authors:** Christophe Thiriet

**Affiliations:** IGDR UMR 6290 CNRS, University of Rennes 1, Campus Beaulieu, Rennes 35000, France

## Abstract

DNA replication occurring in S-phase is critical for the maintenance of the cell fate from one generation to the next, and requires the duplication of epigenetic information. The integrity of the epigenome is, in part, insured by the recycling of parental histones and *de novo* deposition of newly synthesized histones. While the histone variants have revealed important functions in epigenetic regulations, the deposition in chromatin during S-phase of newly synthesized histone variants remains unclear. The identification of histone variants of H3 and unique features of Physarum polycephalum provides a powerful system for investigating *de novo* deposition of newly synthesized histones by tracking the incorporation of exogenous histones within cells. The analyses revealed that the rate of deposition of H3.1 and H3.3 is anticorrelated as S-phase progresses, H3.3 is predominately produced and utilized in early S and dropped throughout S-phase, while H3.1 behaved in the opposite way. Disturbing the expression of H3 variants by siRNAs revealed mutual compensation of histone transcripts. Interestingly, the incorporation of pre-formed constrained histone complexes showed that tetramers of H3/H4 are more efficiently utilized by the cell than dimers. These results support the model whereby the histone variant distribution is established upon replication and new histone deposition.

## INTRODUCTION

Genomic DNA in eukaryotes is associated with proteins to form chromatin. The basic sub-unit of chromatin is the nucleosome, which contains ∼147 bp of DNA is wrapped in about 1¾ turns around a core histone octamer, and a variable region (10–50 bp) of linker DNA (1,2). The histone octamer is composed of a central tetramer of core histones H3/H4 flanked by two heterodimers of H2A/H2B (3). Although the histone proteins within the nucleosome are highly conserved, the existence of histone variants provides structural and functional diversity (4). Indeed, core histone variants are associated with specific chromatin activities, and typically (i) exhibit unique sequence characteristics and timing of synthesis within the cell cycle (5,6), (ii) associate with specialized chaperones (7–10) and (iii) impart distinct biological functions (11).

Isoforms of histone H3 have been identified throughout the eukaryotic kingdom, wherein variants have been associated with specialized chromatin regions. Indeed, the centromeric region of chromosomes exhibit specialized nucleosomes containing the centromeric variant of H3 (CenH3; also known as CENP-A in humans, Cse4 in budding yeast and CID in Drosophila) (11). Even though the centromeric H3 retains several properties of histone H3 and can replace it within the histone octamer, the variant is the most divergent isoform of H3, which affects the nucleosomal structure (12). In addition, the centromeric variant of H3 exhibits a conserved region within the histone fold-domain, so-called CATD, which is essential for the binding of the CenH3 chaperone HJURP and the kinetochore assembly (13). In addition, except in yeast, other isoforms of H3 have been identified. In contrast to centromeric variants of H3, the other isoforms of H3 differ from canonical H3 by only a few amino acids (11). However, a major distinction is that the canonical H3 supplies new histone during DNA replication and is highly expressed during the S-phase of the cell cycle (6). The timely regulated expression of the canonical H3 (H3.1 and H3.2 in mammals, refers as H3.1 hereafter) led to these proteins being defined as replication-dependent variants. The histone chaperone CAF-1 binds to canonical H3/H4 and is involved in the assembly of nucleosomes coupled to DNA synthesis (14). In contrast, the variant H3.3 is expressed at low levels throughout all phases of the cell cycle and can replace the canonical H3.1 at genomic sites undergoing active nucleosome turnover (5). Hence, *de novo* deposition of such histone variants involves dedicated replication-independent histone chaperones (15). The analyses of *de novo* deposition of the H3.3 has led to the identification of two chaperones, HIRA and DAXX (7,9,16).

It is believed that while assembly of nucleosomes containing canonical H3.1 occurs genome-wide during DNA synthesis, the replacement variant H3.3 is enriched in promoters, active genes, and at specific landmarks, such as pericentric heterochromatin and telomeres (10,17,18). Although the genomic distribution of both canonical H3.1 and variant H3.3 has been examined in Drosophila and mammalian cells, how these two newly synthesized isoforms of H3 are utilized during S-phase of the cell cycle remains elusive. Indeed, while parental and newly synthesized histones are assembled into chromatin behind the replication fork, both pools of histones present distinct epigenetic information since parental histones are decorated with post-translational modifications and newly synthesized are almost unmodified (19,20). These differences between parental and newly synthesized histones led to distinguish two pathways of chromatin assembly, the assembly of parental histone, so called the parental histone recycling, and the assembly of newly synthesized histones, so called the *de novo* assembly. Analyses of the recycling of parental H3.1 and H3.3 in human cell lines revealed that the recycling is not equivalent for both variants and is regulated throughout the S-phase. Indeed, it has been shown that H3.3 and H3.1 mark early- and late-replicating chromatin, respectively (21). The analyses of the fate of newly synthesized histones in S-phase requires experimental means to discriminate newly-synthesized histones from parental proteins already residing in the chromatin. Unique features of the slime mold, Physarum polycephalum, including the synchrony of millions of nuclei throughout the cell cycle, and the spontaneous incorporation of exogenous histones which mimics newly synthesized histones, has allowed investigations of defined histone variants and mutants in nuclear import and replication-coupled chromatin assembly during the S-phase of the cell cycle, and provides a tractable system to investigate this question (22–24).

In the present report, the unique features of Physarum have been exploited to investigate how newly synthesized canonical H3.1 and H3.3 variant are utilized during S-phase by the cell. Consistently with expression patterns, a gene encoding the canonical H3.1 that is transcribed exclusively in S-phase and a gene encoding the H3.3 variant that is transcribed throughout the interphase, were identified. Knock-down experiments of the H3 variants have revealed that H3.1 and H3.3 are mutually compensated when their expression is impaired during the S-phase. Exogenous recombinant histones were produced and incorporated at precise times during S-phase into plasmodia, revealing that canonical H3.1 and the variant H3.3 are not uniformly incorporated throughout S-phase. Indeed, the variant H3.3 is mainly *de novo* deposited in early S-phase, but declines over the progression through the S-phase. Conversely, the canonical H3.1 assembly increased throughout the S-phase. Remarkably, significance of the results was emphasized by the correlation between the deposition efficiencies of exogenous histones into chromatin and the amounts individual endogenous transcripts, which is consistent with the model of coordination of the amounts of histones and DNA during S-phase by the transcripts. Furthermore, incorporation of stabilized histone tetramers during the S-phase revealed that under this constrained conformation both the exogenous H3.1 and H3.3 are more efficiently transported in nuclei and *de novo* deposited into Physarum chromatin than the dimeric conformation.

## MATERIALS AND METHODS

### Cultures of Physarum

Physarum polycephalum strain TU291 was maintained in axenic liquid culture as described (25). Naturally synchronous plasmodia were prepared as described (25). The onset of the second mitosis was determined by phase-contrast microscopy observations of smears of tiny explants. All the experiments were carried out during the synchronous cell cycle between M2 and M3.

### Preparation of exogenous histones

The histones used for the incorporation experiments were expressed in *Escherichia coli* BL21 transformed with pET3a plasmid bearing the histone gene. The Physarum histone H3.1 and H3.3 genes were obtained by gene synthesis optimized for *E. coli* (Eurofins), wherein the cysteine residue 110 was substituted with alanine. H3 and H4 were purified together using the procedure described in (26). Histone tetramers containing H3.1(.3)K115C mutation were purified in the same way. The crosslinking of the sulfhydryl with MTS-3-MTS was carried out accordingly to (27). The analyses of the tetramers were carried out by SDS-PAGE in absence of reducing reagent.

### Incorporations into Physarum

For the incorporation of histones into Physarum, plasmodia were cut into fragments of equal size and a solution (10 mM phosphate buffer pH 7.5, 5 mM ATP) containing trace amounts of histone proteins (<1%) was spread on the upper surface. The incorporation was performed as indicated in the figures, the treated plasmodia were kept in growth medium in the dark at 26°C. For the EdU (5-ethyl-2′-deoxyUridine) pulses, 2 μM EdU was added to the culture medium.

For the incorporation of siRNA into Physarum, similarly to protein incorporations, the plasmodia were cut into equal fragments. For each analysis, the two halves from the same plasmodium were treated either with the target siRNA and with the control siRNA, or with the control siRNA and no treatment. The siRNAs were solubilized at a concentration of 100 pmol/μl in 5× siMAX buffer provided by the manufacturer (Eurofins). For the transfection of the siRNAs, 100 μl of 100 nM siRNAs diluted in 1× siMAX in presence of 3 μl lipofectamine RNAiMAX were spread onto the upper cellular surface of the plasmodium fragments. Total RNA were isolated accordingly to (22), following the DNase I treatment, the absence of genomic DNA was control by PCR using 26S genes as target. cDNAs were synthesized using iScript cDNA synthesis kit (BioRad) accordingly to the manufacturer instructions and analyzed using appropriated primer sets, which have been validated for q-PCR (see supplementary table for primer and siRNA sequences). Q-PCR reactions were carried out in triplicates using Maxima SYBR Green qPCR Master Mix accordingly to the manufacturer instructions (ThermoFisher) and the following PCR program [95°C for 10 min, 40× (95°C for 15 s, 60°C for 30 s, 72°C for 30 s) and melting analyses].

### Isolation of nuclei and preparation of chromatin

Plasmodia segments on filter paper supports were washed in 5 mM EDTA. The nuclear fractions were isolated as following: The cells were harvested and disrupted by Dounce homogenization in isolation buffer (28). The cellular material was the pelleted by centrifugation at 700 g for 5 min. The pellet was then resuspended in Percoll-containing isolation buffer (isolation buffer with 25% Percoll) and the suspension was transferred into ultracentrifuge tubes and spun for 40 min at 40 000 g in a Ti 90 rotor. The nuclear fraction was collected in the bottom of the tubes and washed with PBS. Chromatin fraction was prepared from isolated nuclei washed twice in ice-cold buffer (10 mM HEPES pH 7.5, 100 mM NaCl, 3 mM MgCl_2_, 0.5% Triton X-100 and 10% glycerol) and once in ice-cold PBS. Chromatin was then extracted by sonication and the nuclear debris were pelleted at 10 000 g for 5 min. Following chromatin preparations, the analyses were carried out using conventional procedures as described in (26). For the ChIP experiments, the procedure is detailed in (29), and the analyses of precipitated DNA was performed by dot blotting after coupling azido-biotin to EdU in presence of 20 mM sodium ascorbate, 10 mM THPTA and 10 mM CuSO_4_, and revealed with avidin-HRP.

### Fluorescent microscopy and chromatin fiber combing

Fluorescent microscopic observations of nuclei and chromatin fibers were done using a Nikon Ni-E. For the observations of nuclei, smears of Physarum explants were fixed with ethanol, blocked with 5% BSA in PBS-T (PBS with 0.1% Tween 20) for 1h. Then, azido-Alexa Fluo 488 was coupled to EdU in presence of 50 mM sodium ascorbate, 10 mM CuSO_4_ and 1 mM azido-Alexa Fluo 488 for 1h at 37°C. The exogenous histones were detected with anti-FLAG antibody (1/1000) and a secondary antibody coupled to rhodamine (1/500). Chromatin fiber combing was performed as described in (24,30). Briefly, Percoll gradient purified nuclei were resuspended in PBS and spotted in presence of 10 mM EDTA onto silanized microscopy slides treated as described (31). The slides were incubated for 12 min in lysis buffer, and the buffer was removed linearly to extend chromatin fibers at the air/liquid interphase. The fibers were then fixed with 4% formaldehyde in PBS for 10 min ad extracted in permeabilization buffer for 10 min. The slides were then treated as described above for the detection of EdU and exogenous histones.

## RESULTS

### Identification of the H3 family proteins in Physarum

**Figure 1.**
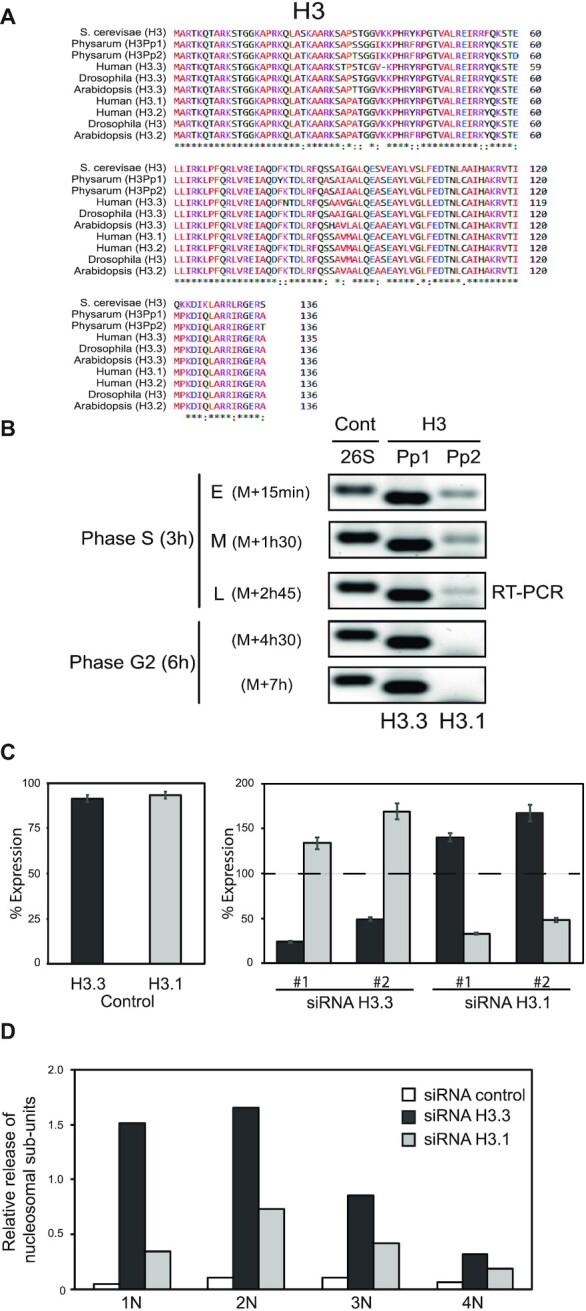
Identification and characterization of the H3 variants of Physarum. (**A**) Alignment of the canonical H3 and the variant H3.3 from a variety of eukaryotes. (**B**) RT-PCR analyses of the expression of the two Physarum H3 variants using as control the 26S rRNA. cDNAs were prepared at specific time throughout the cell cycle, in early (E), mid (M) and late (L) S-phase, and in early and late G2-phase, respectively. The genes identified as H3Pp1 and H3Pp2 correspond to H3.3 and H3.1, respectively. Note that the primers sets used in these analyses were inadequate for quantifications. (**C**) Knock-down analyses of transcripts encoding for H3.3 and H3.1. The control panel corresponds to the percentage of H3.3 and H3.1 cDNA determined by q-RT-PCR, respectively, from cell fragments treated with control siRNA relative to untreated cell fragments. Knock-down analyses were carried out similarly to the control panel, except that cell fragments were treated with specific siRNAs and with control siRNA, and the percentage cDNAs encoding for H3.3 and H3.1, respectively, were determined by q-RT-PCR relative to the control. (**D**) Analyses of chromatin structure following siRNA treatments. Cell fragments were treated with specific siRNAs and control siRNA, respectively. Nuclei were prepared and subjected to MNase treatment (see Supplementary Figure S4 for details). The graph corresponds to the ratio of nucleosomal bands (1N, 2N, 3N and 4N) to undigested DNA.

The exploration of the genome of Physarum led to the identification of three genes encoding for H3 family proteins. The predicted protein sequence comparison showed that two sequences (H3Pp1 and H3Pp2) were nearly identical (∼98% identity) while the third (H3Pp3) was quite divergent (∼53% identity and encoding a predicted protein twice as large). It has been shown that centromeric H3 typically has low homology with other members of the H3 family. I therefore assumed that H3Pp3 might be the centromeric H3 variant of Physarum (Cen-H3) especially since a putative CATD sequence could be identified (Supplementary Figure S1). Regarding the two other members of the H3 family, their high homology suggested that one might be a canonical H3 and the other a less divergent variant H3. However, alignment with orthologue histones failed to distinguish the two (Figure 1A). Thus, to correctly attribute the two proteins, transcription of the genes encoding for H3Pp1 and H3Pp2 was examined at different stages of the cell cycle (Figure 1B). Plasmodia (multinucleated giant cells) were cultured and harvested at defined cell cycle stages, cDNAs were prepared and analyzed by PCR using specific primers. The agarose gel of the amplicons showed that H3Pp1 was transcribed throughout the cell cycle, while, conversely, transcription of H3Pp2 was restricted to the S-phase of the cell cycle. Noteworthy, although the intensity of the H3Pp1 amplicon was greater than that of H3Pp2 amplicon, analyses of these specific sets of primers revealed that they were not appropriated for quantitative PCR. Thus, the comparison the PCR products of H3Pp1 and H3Pp2 is meaningless. However, accordingly to early studies of histone synthesis in cell cultures, the variant H3.3 is produced throughout the interphase, while canonical H3.1 is only synthesized during S-phase (32). Hence, the transcription pattern of H3Pp1 and H3Pp2 genes strongly suggested that they encoded for H3.3 and H3.1, respectively.

It has been shown that in addition to their differential timing of synthesis during the cell cycle, the H3 paralogs differ from some amino-acids in their protein sequence. In contrast to animals, which present differences at position 31 and in residues 87–90 in the N-terminal end of □-helix 2 (human histone) (17), Physarum presents only punctual substitutions at four positions distributed throughout the proteins (T31 versus S31, D59 versus E59, F78 versus Y78 and T135 versus A135 in H3.1 versus H3.3) (Figure S2). These slight divergences between the two H3 paralogs compromised the possibility to detect H3.3 with high specificity using antibodies (33). Furthermore, the composition of a defined culture medium revealed that Lysine is dispensable in Physarum (34), which would hinder accurate SILAC analyses. Thus, to verify whether both H3 paralogs are used during the S-phase of the cell cycle, specific siRNAs were designed and incorporated into Physarum plasmodia for knocking-down the protein expressions during S-phase. Incorporation of a control siRNA revealed a moderate decrease of the amount of mRNAs encoding for H3.3 and H3.1 (<10%) compared to untreated cell fragments (Figure 1C, Control panel), suggesting that the cell fragment treatment presented moderate disturbance. Oppositely, cell fragments treated with specific siRNAs to H3.3 and to H3.1 presented drastic effects on the histone expressions. Indeed, knocking down the expression of either H3 paralog showed a significant decrease of the targeted histone and the over-expression of the untargeted H3. These results strongly suggested that the abundance of H3 is regulated and paralogs are mutually compensated, as it was previously suggested in Tetrahymena (35). Furthermore, the analyses of DNA replication when H3 paralogs are knocked-down failed to reveal significant effect on the incorporation of a Thymidine analog (EdU) (Supplementary Figure S3). Interestingly, these results are consistent with recent studies in yeast, wherein it is shown that the homeostasis of histone concentration and DNA content within the cell is achieved at the transcriptional level (36). MNase digestion patterns of chromatin showed that the knocking-down H3.3 or H3.1 effected the DNA accessibility (Figure 1D, Supplementary Figure S4). These results promote the idea that the newly synthesized H3 paralogs are involved in chromatin organization.

### New H3.3 and new H3.1 are assembled in chromatin in S-phase

**Figure 2.**
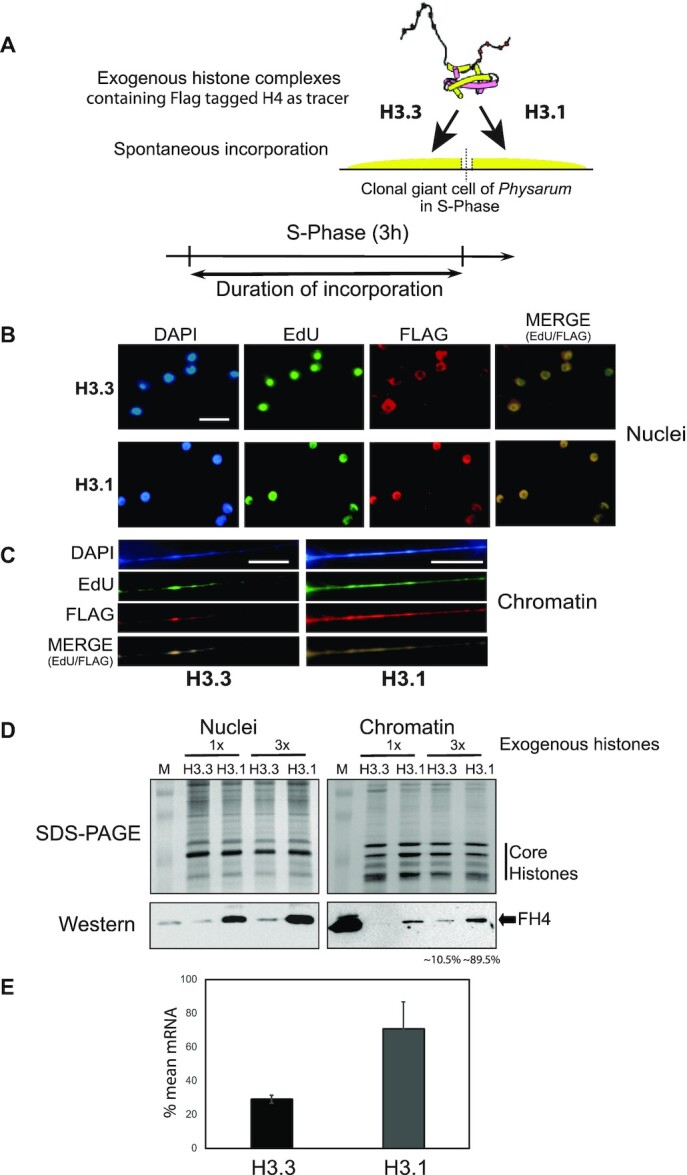
Incorporation of H3.3 and H3.1 containing complexes throughout the S-phase. (**A**) Diagram of the experimental design of incorporation of exogenous histone complexes. (**B**) Exogenous histone complexes are transported into nuclei in S-phase. Following concomitant pulse with EdU and exogenous histone complexes, smears of Physarum were microscopically observed. (**C**) Exogenous histone complexes are assembled into chromatin. Following concomitant pulse with EdU and exogenous histone complexes, chromatin was stretched and microscopically observed. The bars correspond to 10 μm. (**D**) Two different trace amounts (1× and 3×) of exogenous H3.3 and H3.1 are transported in nuclei and assembled in chromatin with different efficiency. Nuclear (Nuclei) and chromatin (Chromatin) fractions were prepared after 3h of incorporation in S-phase. Proteins were resolved in SDS-PAGE and analyzed by western blotting with anti-FLAG antibody. The lanes labeled M correspond to size marker in SDS-PAGE and purified exogenous histones in western blots. (**E**) The genes encoding for H3.3 and H3.1 have different rates of transcription through S-phase. RNAs from different time points in S-phase were isolated and cDNA prepared. Following normalization using 26S rRNA, cDNA samples were mixed and the mean percentage of H3.3 and H3.1 was estimated by q-RT-PCR.

Consistent with the reported expression of the variants of H3 in other eukaryotes (6), both H3.1 and H3.3 are produced during the S-phase in Physarum. Even though the MNase digestion patterns suggested that the newly synthesized H3.3 and H3.1 are involved in chromatin organization, how newly synthesized paralogs are utilized during this stage of the cell cycle has not been elucidated. Certainly, Physarum is ideal for investigating the fate of newly synthesized histones, as it forms plasmodia corresponding to giant cells containing millions of nuclei perfectly synchronous throughout the cell cycle during the vegetative growth (37), and has the unique ability to rapidly uptake exogenous proteins, wherein their fate can be followed within the cell with a fluorescent probe or an epitope tag (26,38,39). Importantly, when exogenous histones are incorporated into the cell, these proteins mimic newly synthesized histones (23,24). Thus, complexes H3.3/FH4 and H3.1/FH4 (FLAG tagged H4) were prepared from recombinant histones and two halves of plasmodia were treated with the same trace amount of exogenous histones at the onset of S-phase and the plasmodium fragments were cultured for the 3 h of the S-phase in presence of the thymidine analogue EdU (Figure 2A). Then, tiny cell explants were smeared onto microscopy slides and analyzed for DNA replication in coupling green Alexa to EdU by click chemistry and exogenous histone incorporation by immunochemical reactions to FLAG epitope (Red labeling) (Figure 2B). As expected for cells in S-phase, microscopic observations of smears revealed EdU staining within all nuclei showing that DNA synthesis occurred during the time frame of the experiments. Immunostaining to detect the FLAG epitope revealed that both exogenous H3.3/FH4 and H3.1/FH4 were localized to the nuclei. However, comparison of smears treated with H3.3 and H3.1 showed distinct patterns of incorporation of the exogenous H3s. Specifically, the staining of H3.3/FH4 was weaker than H3.1/FH4 and, the nuclear staining of H3.3/FH4 excluded the nucleolus, while H3.1/FH4 was more homogenously distributed within the nucleus (Supplementary Figure S5). To determine whether the exogenous histone complexes were incorporated into chromatin, nuclei from cell fragments were isolated and chromatin fibers were stretched onto microscopic slides (Figure 2C). The slide staining revealed, as expected, the incorporation of EdU into genomic DNA. The immunostaining of the exogenous histones co-localized with DNA and coincided with DNA synthesis, suggesting that the assembly of both H3.3 and H3.1 in chromatin during S-phase is coupled to replication.

To gain insights into the extent of incorporation of H3.3- and H3.1-containing complexes into nuclei and chromatin, plasmodium fragments were treated with two amounts of FLAG tagged exogenous histones (1× and 3×). Nuclear and chromatin fractions were prepared and analyzed by western blotting (Figure 2D). The results showed that the FLAG signal intensities of exogenous histones in nuclei and in chromatin were proportional to the amounts of proteins spread onto the cellular surfaces, meaning that quantities of exogenous histones did not saturate the biological processes of nuclear import and chromatin assembly. Furthermore, whereas similar amounts of exogenous histones were spread onto the cellular surfaces at the onset of the S-phase, after the 3h of S-phase H3.1/FH4 was ∼9-fold more abundant in nuclei and in chromatin than the H3.3/FH4. These results were consistent with the microscopic observations, and suggested a preferential *de novo* deposition of canonical H3.1 during the S-phase. It has been shown in yeast that the histone protein concentration was coordinated by the amount of transcripts (36). Hence, to verify whether such coordination was also validated in Physarum, quantification of mRNAs encoding for H3.3 and H3.1, respectively, were carried out (Figure 2E). cDNAs from plasmodia harvested through the S-phase were prepared, normalized with 26S and analyzed by q-RT-PCR with primers specific for H3.3 and H3.1. The results showed that the cumulative amount of H3.3 and H3.1 at the time points when the cell fragments were harvested is lower for H3.3 (∼30%) than H3.1 (∼70%). Therefore, the difference of exogenous histones utilized by the cell through the S-phase correlated with the abundance of mRNA of H3.1 and of H3.3 at the same stage of the cell cycle.

### H3.3 and H3.1 display distinct patterns of usage through the S phase

**Figure 3.**
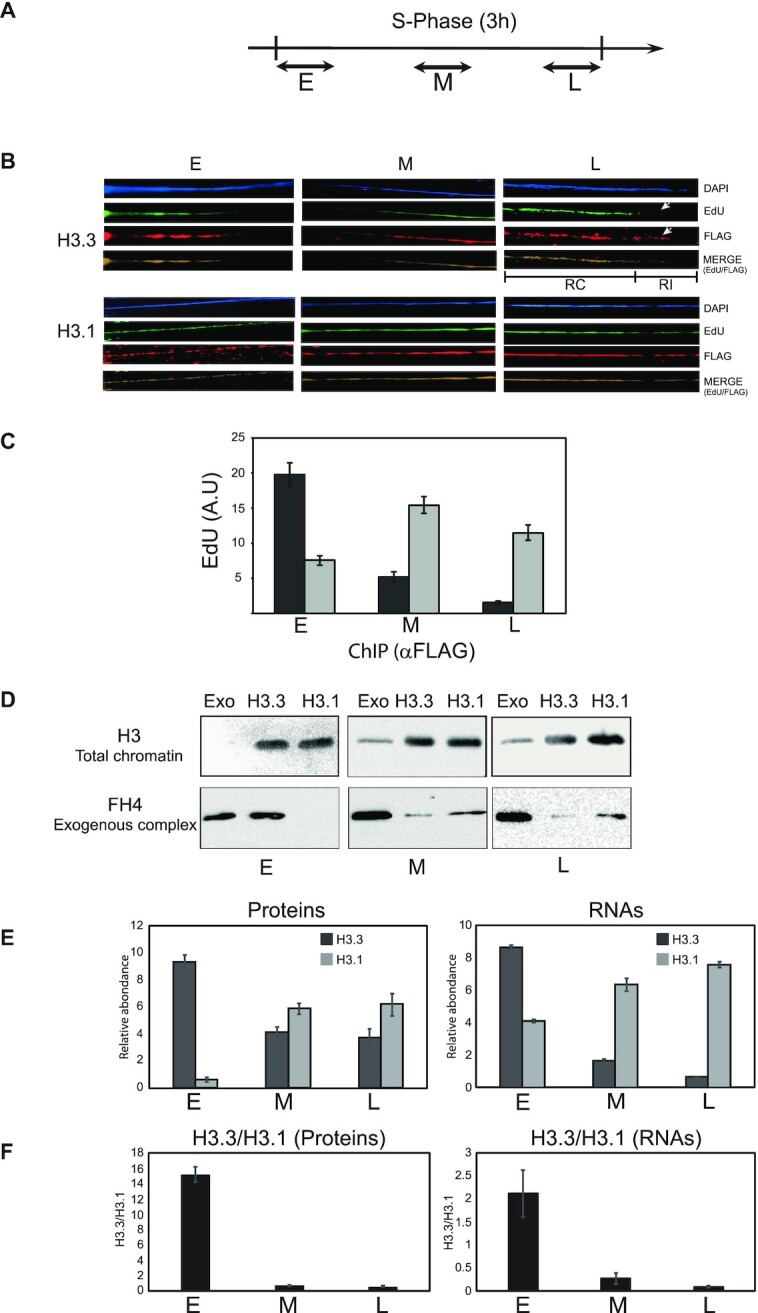
The H3 variants exhibit distinct patterns of usage at specific stage of the S-phase. (**A**) diagram of the incorporation of exogenous histone complexes. Exogenous histones are incorporated for 30 min in early (E), mid (M) and late (L) S-phase. (**B**) Exogenous histones are assembled into chromatin. Microscopic observations of chromatin stretches as (Figure 2C). H3.3- and H3.1-containing complexes are assembled at precise moments of S-phase. RC and RI correspond to replication-coupled and replication independent assembly, respectively. (**C**) Estimation of the amount of replicating DNA associated with exogenous histones. Exogenous histones were incorporated as in (B) and chromatin-containing exogenous histones was precipitated using anti-FLAG magnetic beads. Replicating DNA containing EdU was coupled with biotin and quantification was performed by dot-blots. (**D**) Following the incorporation of exogenous histone complexes in early (E), mid (M) and late (L) S-phase, chromatin fractions were prepared and analyzed by western blotting. The blots were immuno-revealed with anti-H3 (H3) to control the amount of chromatin and with anti-FLAG (FH4) to estimate the amount of exogenous histone complexes. The lanes Exo correspond to purified exogenous histone samples. (**D**) Quantifications of exogenous histones and specific RNAs in E, M and L S-phase. Exogenous histones were quantified relative to endogenous H3 (proteins). Specific H3.3 and H3.1 transcripts were quantified by qRT-PCR, taking as control 26S RNA. (**E**) Ratio of H3.3/H3.1 of exogenous proteins and endogenous RNAs. The ratios were calculated from western blot analyses (proteins) and from qRT-PCR (RNAs).

Studies in *S. cerevisiae* and Drosophila cells have reported that chromatin landscape following replication is governed by transcription (40,41). Pioneer work in Physarum has shown a physical relationship between replication and transcription in early S-phase (42). Indeed, electron microscopic observations of chromatin spread derived from early S-phase revealed nascent transcripts on both strands of replication bubbles. Furthermore, transcription in early S-phase was detected only if DNA synthesis was not inhibited. Since all nuclei are perfectly synchronous within a Physarum plasmodium allowed to determine whether *de novo* assembly of the H3 paralogs was regulated through the S-phase. To ensure that the analyses are not biased by different efficiencies of incorporation of the two exogenous histone complexes, kinetics of incorporation of similar amounts of proteins into nuclei in mid S-phase was examined. The quantifications of western blots revealed that both exogenous H3.3 and H3.1 complexes accumulated into nuclei with similar rates, showing therefore that the histones complexes present comparable efficiencies of incorporation (Supplementary Figure S6). Thus, tracer amounts of exogenous H3/FH4 complexes containing the two H3 variants were incorporated into Physarum plasmodia at early S-phase (0–30 min), mid S-phase (1 h 15 min to 1 h 45 min) and late S-phase (2 h 30 min to 3 h), along with EdU to label newly synthesized DNA at each time point (Figure 3A). Nuclei from each cell fragment were isolated and chromatin was spread onto glass slides for analysis (Figure 3B). The fluorescent microscopic observations revealed that during the periods of incorporation throughout S-phase, both exogenous H3.3 and H3.1 were detected in chromatin. However, differences could be observed between the two H3 variants. First, the exposure times required to detect exogenous histones throughout the S-phase suggested the rate of *de novo* deposition of the two H3 variants changed over time and was anticorrelated as S-phase progressed. Indeed, the exposure times increased through S-phase for detection of H3.3 (from 100 ms in early S to 1000 ms in late S), suggesting the rate of incorporation of this variant was highest in early S then dropped significantly as S-phase progressed. However, exposure times for H3.1 shortened through S-phase (from 1500 ms in early S to 80 ms in late S), suggesting the rate of incorporation increased throughout S-phase. Remarkably, *de novo* deposition of H3.1 coincided with DNA synthesis through S-phase, this was also observed in early S for H3.3, but in late S, *de novo* deposition of H3.3 was also detected in non-replicating regions. Thus, at least in late S-phase, H3.3 is *de novo* deposited via replication- coupled (RC) and replication-independent (RI) mechanisms.

The microscopic analyses of chromatin spreading suggested different utilizations of the newly synthesized H3 variants through S-phase. However, the irregular DAPI staining along chromatin fibers suggested that chromatin structures were not completely released, and thus, did not allow accurate quantifications of exogenous histones associate with replicating DNA. Hence, to prevent chromatin structures in quantitative analyses, ChIP was carried out using anti-FLAG antibody to precipitate exogenous histones and replicating DNA was evaluated by dot-blots of EdU containing DNA (Figure 3C). The results showed that H3.3 associated preferentially with replicating DNA in early S-phase, while H3.1 replication-coupled chromatin assembly was predominant in mid and late S-phase. Next, the analyses of de novo deposited exogenous histone complexes were carried out by incorporation of FLAG tagged exogenous histone complexes at different time points of S-phase followed by chromatin preparations and western blotting (Figure 3D). Blots revealed with H3 antibodies, which detected both endogenous and exogenous (<1%) histones showed comparable amounts of chromatin from cell fragments treated with either exogenous histone complexes. In contrast, detection of exogenous histone complexes using FLAG epitope carried by exogenous H4 (FH4) revealed that the exogenous H3.3-containing complex is mainly deposited in chromatin in early S-phase and is reduced in mid and late S-phase. Oppositely, exogenous H3.1-containing complex is almost undetectable in early S and the signal raise over S-phase progression.

To gain insight into how the production of the two isotypes of H3 in S-phase relates to their relative rates of deposition, the transcription of the genes encoding for each H3 was examined (Figure 3E). Quantifications of western blots and q-RT-PCR at specific time points of the S-phase showed that H3.3 *de novo* deposited in chromatin and mRNA are maximal in early S-phase. In contrast, mRNA and *de novo* deposition of H3.1 are minimal in early S-phase. Therefore, the correlation between the amounts of mRNAs and the amounts of exogenous histones *de novo* deposited into chromatin showed that the synthesis and the utilization of the two variants of H3 are tightly regulated during the S-phase and suggested that the amounts of H3 paralogs through genome replication are coordinated at the transcription level as shown in yeast (36). To verify the link between the novo deposition of H3 variants and the amounts of mRNAs the ratios of H3.3 to H3 proteins and mRNAs were calculated (Figure 3F). Although the trends were similar between the two graphs, reflecting a correlation between protein and RNA amounts, the close examination of the values of the ratios revealed, however, that the protein ratio was ∼8-fold greater than that of RNAs in early S-phase and ∼2.5- and ∼7.5-fold in mid-S-phase and late S-phase, respectively. These changes in the ratios between proteins and RNAs through S-phase suggested that additional level of regulation might be involved.

### *De novo* deposition of the H3 isotypes is regulated by the formation of tetramers

The most obvious mechanism of regulation of the amount of newly synthesized H3 variants deposited into chromatin involved a regulation of the amount of chaperones responsible of the histone assembly. Genes encoding for HIRA and CAF-1A were identified in the Physarum genome and their transcription at specific time points of S-phase was examined by q-RT-PCR (Supplementary Figure S7). The results revealed that the amounts of HIRA and CAF-1A transcripts involved in the assembly H3.3 and H3.1, respectively, did not significantly fluctuate through S-phase. Thus, even though chaperones are involved in *de novo* deposition of the H3 variants, the transcript abundance at different time points of S-phase suggested that the amount of histone chaperones is not involved in the regulation of the amount of histones variants assembled throughout S-phase. A putative regulation at the nuclear import level of the H3 paralogs is unlikely, as both variants share the same mechanism of nuclear import (8).

**Figure 4.**
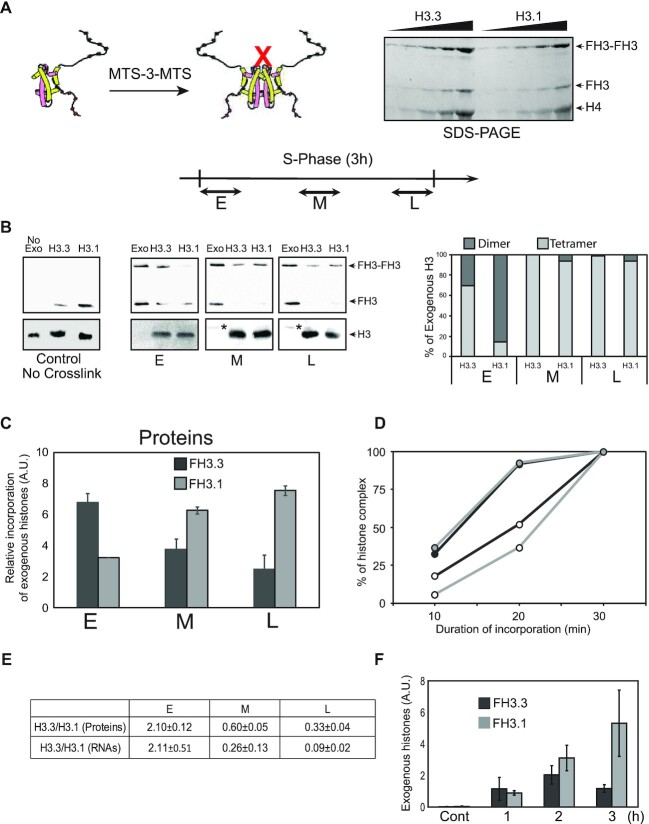
Exogenous H3.3 and H3.1 tetramers rather than dimers are preferentially utilized by the cell through the S-phase. (**A**) Diagram of the formation of the tetramers and SDS-PAGE of the cross-linked proteins. Diagram of the incorporation of exogenous proteins for 30 min in early (E), mid (M) and late (L) S-phase. (**B**) Western blot analyses of the incorporation H3.3C155- and H3.1C115-containing complexes in chromatin. (Control no crosslinking) panel corresponds to the incorporation throughout S-phase of mutant (K115C) exogenous histones without crosslink. The first lane (no Exo) showed the specificity of the analyses in cell fragment untreated with exogenous histones. The other blots correspond to the incorporation of exogenous histone complexes in early (E), mid (M) and late (L) S-phase, and the analyses of chromatin fractions by western blotting. The blots were immuno-revealed with anti-H3 (H3) to control the amount of chromatin and with anti-FLAG (FH3 dimers and FH3-FH3 tetramers). The lanes (Exo) correspond to purified exogenous complexes. Note that the stars correspond to the FH3 in the lanes of purified exogenous complexes. The graph represents the percentage of tetramer (light grey) versus dimer (dark grey) assembled in chromatin. (**C**) Quantifications of exogenous histones in E, M and L S-phase. Exogenous histones were quantified relative to endogenous H3. (**D**) Tetramer and dimer conformations of H3/H4 are *de novo* deposited into chromatin with different rates. The deposition into chromatin of exogenous histones was examined after 10, 20 and 30 min respectively. Western blots were quantified for each exogenous complex, taking as 100% the amount of individual complex. Filled circles correspond to tetramers and empty circles correspond to dimers, respectively. Dark lines correspond to H3.3 and light lines correspond to H3.1, respectively. (**E**) Table of the calculation of the ratio H3.3 to H3.1proteins in E, M and L S-phase, and the ratio H3.3 to H3.1 RNAs obtained from Figure 3F. (E) Analyses of the accumulation of exogenous complexes through the S-phase. Exogenous histones were incorporated at the onset of the S-phase and nuclear fractions from plasmodium fragments were prepared at the onset of the S-phase (Cont), after 1, 2 and 3 h of incorporation, respectively. Exogenous histones were quantified from western blots.

To shed light on the mechanism of the regulation of *de novo* deposition of the H3 variants during the S-phase beyond the synthesis of the histones, I postulated that this regulation might occur at histone complex conformation level. It is well-established that nucleosome is composed of a central tetramer of H3/H4 (3). However, whether *de novo* deposition of H3/H4 involves the formation of the tetramer prior to the loading onto DNA is still under debate. Since Physarum presents the unique characteristic of spontaneous internalization of exogenous proteins, this model system is well-suited for examining whether the H3/H4 tetramer is deposited in chromatin. Thus, H3 and H4 in their tetrameric conformation was prepared by using the method developed by Bowman *et al.* (27), which consists in crosslinking two H3s at cysteine substituted residues 115 with MTS-3-MTS (Figure 4A). Notably, after crosslinking a mixture of monomer and dimer of the H3 variants were detected in SDS-PAGE. Thus, to prevent misinterpretations of the usage of dimers versus tetramers, the FLAG epitope was transferred to the variants of H3, wherein FLAG tagged cross-linked H3 (FH3-FH3) and FLAG tagged H3 (FH3) could be unambiguously resolved in SDS-PAGE. As a control, uncross-linked complexes containing one or the other of H3 C115 paralogs were incorporated through S-phase to determine whether disulfide bonds formed between exogenous complexes through the experimentation, which lacks the presence of sulfhydryl reagent throughout the procedure. Chromatin was then prepared and analyzed by western blotting in absence of reducer (Figure 4B, control no crosslink blots). As expected, the results showed that *de novo* deposition of the H3 variants tagged on the H3 proteins was very similar to what was observed with FLAG tagged H4 (see Figure 2D), demonstrating no effect of the FLAG position within the histone complexes. Moreover, the immune-detection of the FLAG epitope revealed the presence of bands corresponding to monomers of FH3.3 and FH3.1 and failed to detect dimers resulting of the formation of spontaneous disulfide bonds between exogenous H3s. Hence, mixtures of FLAG tagged tetramers and dimers (∼1:1) of H3.3 or H3.1 were incorporated in early, mid and late S-phase, respectively. Chromatin fractions were prepared and analyzed by western blotting (Figure 4B, E, M and L blots). Clearly, the detection using anti-H3 antibodies (H3) showed that the amounts of chromatin from cell fragments treated with H3.3 and H3.1 complexes were comparable. Moreover, note that exogenous FLAG-tagged H3s, which were barely seen in the Exo lanes (see stars in M and L panels) but were not detected in chromatin fractions demonstrating trace amounts of exogenous histones. Next, stripped blots were analyzed with anti-FLAG antibody to determine which conformation (dimer and tetramer) was utilized by the Physarum cell in S-phase. Given that mixtures containing both conformations (dimer and tetramer) were incorporated, within the same lane, the signal corresponding to dimer (FH3) and tetramer (FH3-FH3), respectively, is the reflection of newly synthesized histone complex utilized by the cell for *de novo* deposition. These analyses showed that the exogenous histone tetramers are deposited *de novo* in chromatin, meaning that tetramers are transported into nuclei and then assembled in chromatin. The quantifications of the blots showed that except in early S-phase the vast majority of the exogenous complexes utilized by the cells are tetramers. In early S-phase, both dimer (∼30%) and tetramer (∼70) of H3.3/H4 are assembled in chromatin, while tetramer (∼85%) of H3/H4 represents the majority of *de novo* H3 deposited. Importantly, while the experimental data exhibited preferential usage of tetramer conformation of both H3 paralogs in the time frame of the analyses through S-phase, it cannot be excluded that the reduced efficiency of *de novo* deposition of dimer conformation corresponds to the requirement for one dimer to associate with another. To test this hypothesis, mixtures of histone complex conformations were spread onto the cellular surface of plasmodia, and cell fragments were harvested after 10, 20 and 30 min, respectively, followed by chromatin preparation and western blotting analyses. The calculations of the percentage of individual complex over time was then reported onto a graph for estimating the slopes of *de novo* deposition of the different complex conformations (Figure 4D). These analyses showed that between 10 and 20 min, the slopes of tetramer conformation for both H3 paralog complexes were about twice greater than those of dimer conformations (∼0.57 versus ∼0.32). These trends are however inverted beyond 20 min since almost all tetramers were already deposited into chromatin (∼0.08 versus ∼0.55). Altogether, these data indicated that as long as exogenous tetramer is available, its *de novo* deposition in chromatin is faster than exogenous dimer. However, when the exogenous tetramer pool is exhausted the efficiency of dimer deposition raises, suggesting that two dimers need to associate together for being *de novo* deposited into chromatin.

To determine whether constraining the H3/H4 complexes to be in a tetrameric conformation was readily a novel of regulation in the coordination of histone proteins by their RNA during S-phase, ratios of exogenous histone paralogs at the different time points of S-phase and compared to those of RNAs. Clearly, the ratios exhibited similar ranges in early S-phase, but for the other time points, the histone protein and RNA ratios revealed some divergences, the ratios of proteins in mid and late S-phase are ∼2 fold and ∼4 fold higher, respectively, compared to the RNAs ratios. This indicated that the tetrameric conformation is a level of regulation in early S-phase, but this conformational regulation of histone complexes is reduced as S-phase progresses, and therefore, additional processes might be involved.

It is well-established that nucleosomes are dynamic and involved histone turnover and replacement. Possibly, the fate of the H3 variants within the replicating nuclei might be involved in histone homeostasis implying that H3.3- and H3.1-containing histone complexes exhibit distinct fates in nuclei. This was determined by the incorporation of the exogenous histone complexes at the onset of S-phase and their accumulation in nuclei was examined by western blotting after 0, 1, 2 and 3 h, respectively. Clearly, these analyses showed that the fates the two H3 variants diverged over S-phase. Indeed, while for the first hour both H3 paralogs presented comparable accumulations in nuclei, gradual discrepancies of exogenous histone variant accumulation in nuclei were observed after 2 and 3 h. Indeed, while nuclear exogenous H3.1-containing complex gradually accumulated as S-phase progressed, loss of exogenous H3.3-containing complex was detected between 2 and 3 h of S-phase. Altogether, the results demonstrated different fates of H3.3 and H3.1 through S-phase, suggesting a fine tuning of histone variants for the maintenance of genome integrity throughout replication that might be coordinated by transcription.

## DISCUSSION

The replication of the genome occurring during the S-phase of the cell cycle involves the assembly of newly synthesized histones to re-establish the packaging of DNA and the epigenetic information. The present study investigated the usage of H3 paralogs during the S-phase of the cell cycle. The analyses have taken advantages of peculiar features of the slime mold, Physarum polycephalum, as the synchrony of millions of nuclei contained within a single plasmodium and the ability for this model system to internalize exogenous histones, which perfectly behave as newly synthesized histones (37). It is generally believed in cycling cells that canonical histone H3.1 synthesis is restricted to S-phase, while the variant H3.3 is produced throughout the interphase (6,32). In Physarum, the analyses of histone gene transcription revealed that the two H3 genes are transcribed in S-phase and only one is transcribed in G2-phase (Figure 1C). These results are consistent with the analyses of histones syntheses detected by pulses of radio-active amino acid, wherein it was shown that ∼95% of histones are synthesized during S-phase (43,44).

The paralogs of H3 have been also defined by the mechanism of their assembly in chromatin. Hence, replication-coupled chromatin assembly (RC) is associated with H3.1 and replication-independent chromatin assembly (RI) refers to H3.3. Although these definitions are verified when the synthesis of one or the other H3 variant is exclusive of the cell cycle stage, for H3.1 during the S-phase and for H3.3 outside this cell cycle stage (6,32), the usage of newly synthesized H3.1 and H3.3 during S-phase has not been completely elucidated. It has been documented that H3.3 is enriched at specific chromosomal landmarks, as at transcriptionally active regions, pericentric heterochromatin and telomeres (11,45). However, deposition of newly synthesized H3.3 in S-phase has been only reported at centromeres (46). These data contrasted with those of parental histone recycling showing that parental H3.3 and H3.1 are preferentially transferred to replicating DNA in early and late S-phase, respectively (21). It has been shown that incorporation exogenous histones into Physarum cells mimicked newly synthesized histones and make possible to examine their fate using an epitope tag or a fluorescent labeling (26,38,39). Using this experimental strategy, it was possible to determine the fate of exogenous H3 paralogs in S-phase, which mimicked newly synthesized H3.3 and H3.1, respectively. The data indicated that the efficiency of *de novo* deposition of both H3 paralogs are anticorrelated throughout the S-phase. New H3.3 is preferentially utilized in early S-phase and decreased over time, while H3.1 *de novo* deposition increased as S-phase progressed (Figure 3). Interestingly, the patterns of usage of newly synthesized H3 variants examined in the present report corresponded to the distribution of the recycling of parental histone variants in HeLa cells relative to replication timing (21). The correlation of the timing within the S-phase of recycling and *de novo* deposition of H3 paralogs indicated that the usage of newly synthesized H3 variants is involved in copying the chromatin landscape after the doubling of DNA and contribute to the epigenetic inheritance from one generation to the next one. Remarkably, it has been shown in budding yeast and Drosophila cells that, rapidly after replication factors involved in transcription bind to DNA and promote remodeling of the chromatin landscape (40,41). The present study reinforces the co-ordination between replication and transcription as active genes are found to replicate early, and correlates with the deposition of newly synthesized H3.3. These results suggested that the occupancy of H3.3 within the chromatin landscape is copied during replication, which should ensure the chromatin landscape inheritance to the next cell cycle after mitosis. Beyond *de novo* deposition of H3.3 in early S-phase, chromatin fiber stretching and the accumulation of the variant in nuclei showed that at least in late S-phase (Figures 3 and 4), the histone H3.3 presented a high rate of turn-over and its deposition partly involved replication-independent process. This suggested that two mechanisms involving H3.3 occur during S-phase, corresponding to replication-dependent *de novo* deposition (RC) and replication-independent replacement (RI), respectively.

Recently, it has been shown in budding yeast that the amount of histone proteins is coupled to the DNA content indicating the existence of a coordination that is achieved at the transcription level (36). The experiments of knock-down of H3 paralogs in Physarum showed that DNA replication is not impaired, but transcription of histone genes revealed a mechanism of mutual compensation of the targeted variant by the untargeted one (Figure 1C). This suggested that similarly to yeast, in Physarum, histone transcripts coordinated the amount of histones and DNA replication. Such mechanism of coordination by transcripts might also interest other organisms other than yeast and Physarum as suggested by experiments of transfection of histone genes avoiding toxic effect of over-expression. Although the coordination of the amount of histones by the transcripts might be a general mechanism for histone homeostasis, the present work showed that the conformation of the histone complexes is another level of the fine tuning between histone synthesis and DNA replication. Early studies have proposed that H3/H4 forms stable tetramers and are not dissociated over several cell cycle (38,47). However, more recently, other analyses refuted this model as suggested by the association of H3/H4 dimer with deposition complexes leading to the assembly in chromatin of two H3/H4 dimers (8). So far, whether H3/H4 is utilized by the cell under tetrameric or dimeric conformation is still under debate (8,47). The data in the present report provide new insights into the usage of dimeric and tetrameric H3/H4 complex conformations in H3/H4 *de novo* deposition. Indeed, the incorporation of a mixture of constrained H3/H4 tetramer and H3/H4 dimer in Physarum cells allowed to directly examine the fate of the histone complexes. Unambiguously, the results showed that *de novo* deposition is promoted by the conformation of the histone complexes, as H3/H4 tetramers are deposited into chromatin more efficiently than dimers (Figure 4). Furthermore, since *de novo* deposition in chromatin of H3/H4 in dimeric conformation occurred at lower rate than tetramer suggested that two dimers need to associate together to end up into chromatin. It will be interesting in future works to determine whether the tetrameric conformation required for efficient *de novo* deposition, which results of the association of two dimers is promoted by the histone chaperones as suggested by the association of CAF-1 with H3/H4 dimer (48,49).

## DATA AVAILABILITY

All data needed to evaluate the conclusions in the paper are present in the paper and/or Supplementary Data.

## Supplementary Material

gkac060_Supplemental_Files
