# Supplementary material for: Usage of the H3 variants during the S-phase of the cell cycle in Physarum polycephalum
Source: Nucleic Acids Res. 2022 Feb 7;50(5):2536–48. doi: 10.1093/nar/gkac060 (PMC8934661; doi:10.1093/nar/gkac060)
Supplement: gkac060_Supplemental_Files [file gkac060_supplemental_files.zip › Figure Supp revised.ai.pdf]

Centromeric H3

```
Drosophila (CID)      MPRHSRAKR-APRPSANNSKSPNDDDTAFR-------SPEPEDGTDYGLEFTTS----QL 48
Arabidopsis (Cen-H3)  ------------------------------------------------------------ 0
S. ceverisae (Cse4)   --------------MS--------SKQQWVSSA-IQ-----SDSSGRSLSNVNRLAGDQQ 32
Human (CENP-A)        ------------------------------------------------------------ 0
Physarum (Cen-H3)     MPRFNHPPKNARKPSSPKRKEPSKVTDTFSKTAAVRQSPRKKPAQNYNYSNNNNNRGRSP 60

Drosophila (CID)      TLQDNN--------------------------RRSSTLRRDAGR---------------- 66
Arabidopsis (Cen-H3)  -------------------------------------MARTKHR--------VTRSQPRN 15
S. ceverisae (Cse4)   SINDRALSLLQ-------RTRATKNLF----------PRREERR-RYESS-KSDLDIETD 73
Human (CENP-A)        ------------------------------------------------------------ 0
Physarum (Cen-H3)     TRSDRDRSISRSRSRSKSPTRPTKKPRPAPTSTRSPSPSRSRSRSRSRSPTRPAKKPHTS 120

Drosophila (CID)      --RQPAARDSS----------TSGEEEDQENR----YP-TTRSPQTRRMTVQQESKTRAA 109
Arabidopsis (Cen-H3)  QTDAAGASSSQAAGPTTTPTRRGGEGGDNTQQT---NP--TTSPATGTRRGAKR--S--- 65
S. ceverisae (Cse4)   YEDQAGNLEIETE----------NEEEA-EMETEVPAPVRTHSYALDRYVRQKR--R--- 117
Human (CENP-A)        ------------MGPRR----RSRKPEAPRRR----------SPSPTPTPGPSR--R--- 29
Physarum (Cen-H3)     AKTVPGTSRSRSKSPTR----PAKKPAASSSSSAPKSPVRTKKASSSHTPVGQK--T--- 171
                                              :                 .         ..

Drosophila (CID)      GPVAAQNQTRRRKAANPMSRAKRMDREIRRLQHHPGTLIPKLPFSRLVREFIVKYS-DDE 168
Arabidopsis (Cen-H3)  RQAMPRGS---QKKSYRYRPGTVALKEIRHFQKQTNLLIPAASFIREVRSITHMLA-PPQ 121
S. ceverisae (Cse4)   EKQRKQSL---KRVEKKYTPSELALYEIRKYQRSTDLLISKIPFARLVKEVTDEFTTKDQ 174
Human (CENP-A)        GPSLGAS------SHQHSRRRQGWLKEIRKLQKSTHLLIRKLPFSRLAREICVKFT-RGV 82
Physarum (Cen-H3)     RPPIGIVT---PSRKHKFRPGTRCLMEIRRFQKSTDLLLRKLPFARLVKEVSDMFS-K-E 226
                                                 ***: *:    *:     * * .:..    :

Drosophila (CID)      PLRVTEGALLAMQESCEMYLTQRLADSYMLTKHRNRVTLEVRDMALMAYICDRGRQF-   225
Arabidopsis (Cen-H3)  INRWTAEALVALQEAAEDYLVGLFSDSMLCAIHARRVTLMRKDFELARRLGGKGRPW-   178
S. ceverisae (Cse4)   DLRWQSMAIMALQEASEAYLVGLLEHTNLLALHAKRITIMKKDMQLARRIRGQFI---   229
Human (CENP-A)        DFNWQAQALLALQEAAEAFLVHLFEDAYLLTLHAGRVTLFPKDVQLARRIRGLEEGLG   140
Physarum (Cen-H3)     PFRWQGKAILAVQEATEIFMVRLFEDANLCAIHAKRVTIMPKDIQLARRIRGPES---   281
                        .    *::*:**: * ::.   : .: : : *  *:*:  :*.  *   : .
```

CATD

Fig.S1: Alignement of centromeric H3 from a variety of eukaryotes. The underline Physarum sequence corresponds to the CATD region.

PpH3.1 ARTKQTARKS TGGKAPRKQL ATKAARKSAP TSGGIKKPHR FRPGTVALRE IRRYQKSTDL LIRKLPFQRL
VREIAQDFKT DLRFQASAIA ALQEASEAYL VGLFEDTNLC AIHAKRVTIM PKDIQLARRI RGERT

PpH3.3 ARTKQTARKS TGGKAPRKQL ATKAARKSAP SSGGIKKPHR FRPGTVALRE IRRYQKSTEL LIRKLPFQRL
VREIAQDYKT DLRFQASAIA ALQEASEAYL VGLFEDTNLC AIHAKRVTIM PKDIQLARRI RGERA

Fig.S2: Sequences of Physarum histone H3.1 and H3.3. the highlighted residues indicated the punctual alterations between H3.1 and H3.3 that distributed throughout the protein sequences.

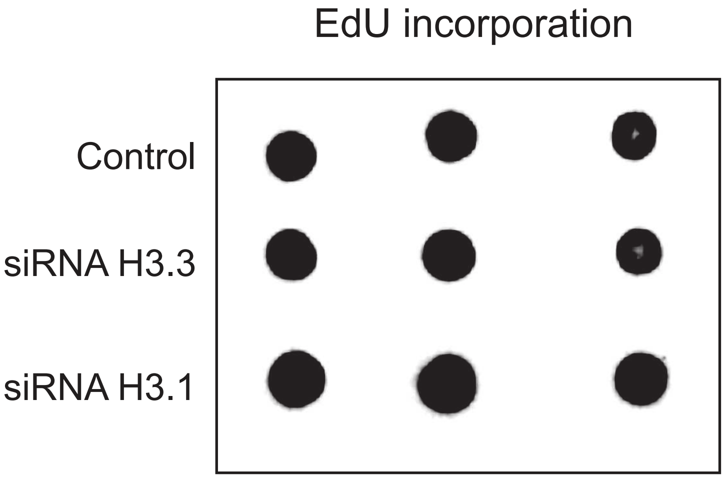

Fig.S3 Knock-down of H3.3 and H3.1 did not affect DNA replication. Dot-blots were carried out to detect EdU incorporation into DNA concomitantly with siRNA treatments.

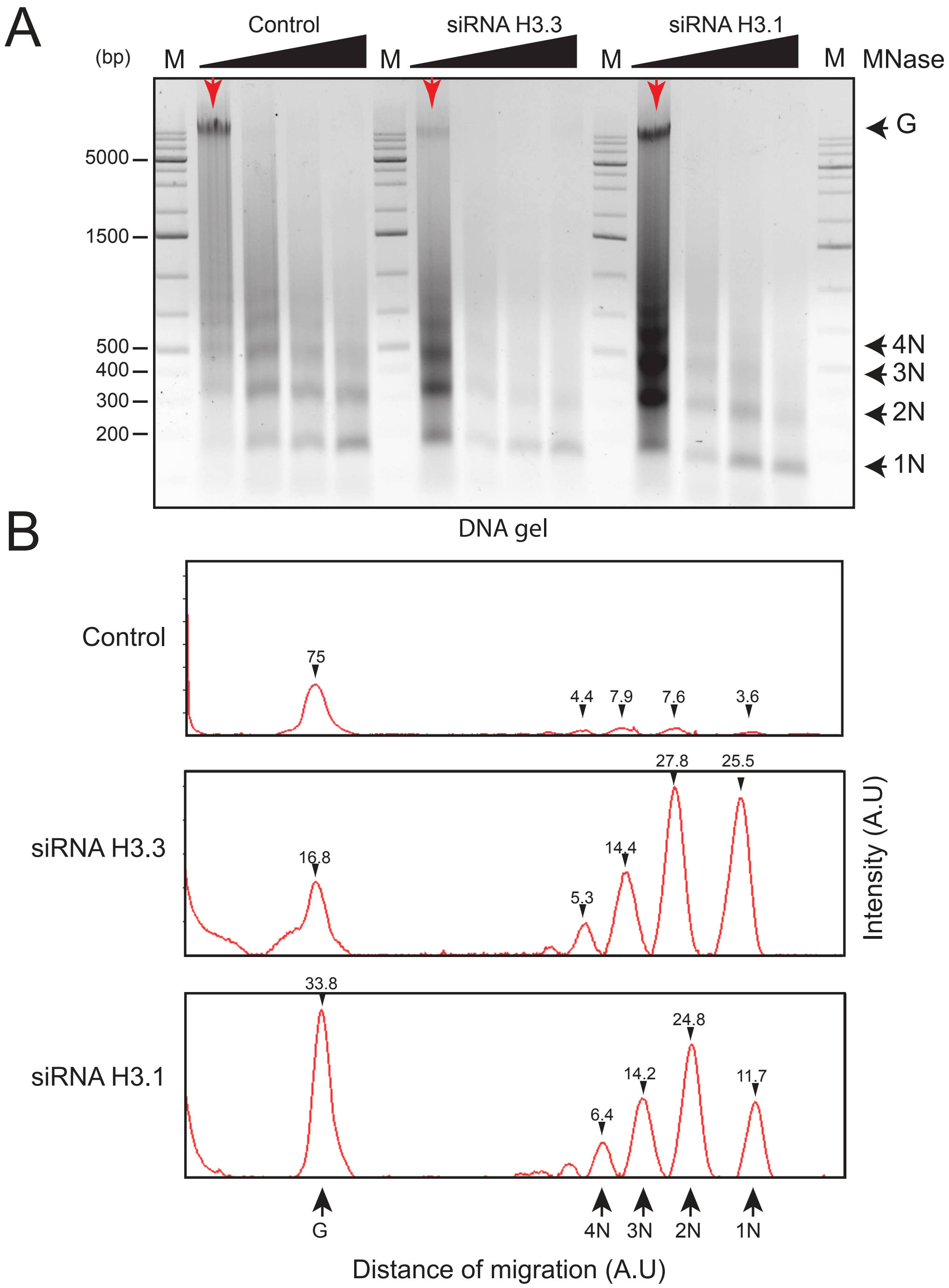

Fig.S4: H3 knock-down affected chromatin structures. (A)Following siRNA treatments, nuclei were isolated and chromatin was digested with 0.15U of MNase for 1, 2, 4 and 6 min respectively. Digested DNA was then analyzed by agarose gel electrophoresis stained with ethydium bromide. (B) Shown are the DNA profils of the MNase digestions after 1min. The value indicated above the peaks correspond to volume of the respective bands determined using biorad software.

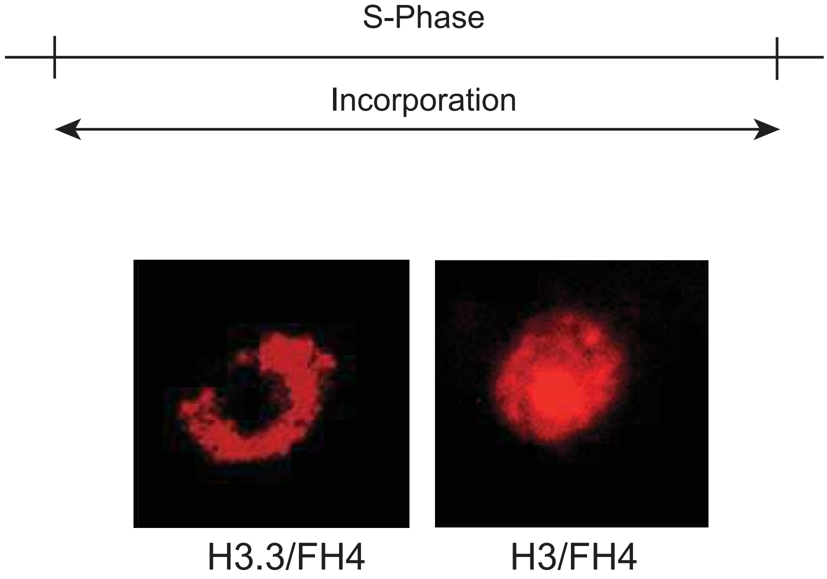

Fig.S5 Nuclear distribution of exogenous histones: H3.3/FH4 and H3/FH4 were incorporated at the onset of S-phase. Cell fragments were smeared at the end of S-phase and microscopically visualized following appropriated treaments.

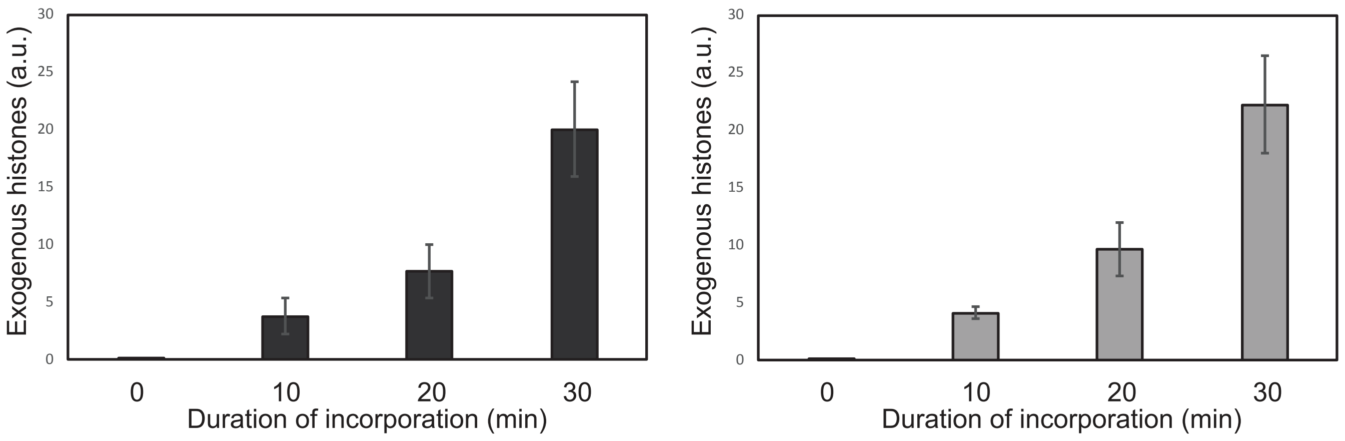

Fig.S6 Rate of incorporation of H3 variants: H3.3/FH4 (dark grey) and H3/FH4 (light grey) were incorporated in mid-S-phase . Nuclei were isolated after defined duration of incorporation and incorporation was estimated by Western blotting. Note that the two graphs resulted from indiviual analyses.

CAF-1A

M D A W L S I Y L C L V W A V F M D A W L K T K R V N T P Q N D V N N A P A P I T D N M D I D T T P K Q E D T K I K S P S K T K S P S K P L P P D
L Q N C F V L L S P C P T F P P T Q N T P P P H P N I P N G N T L Q D K P I L V D P S V N Q S H A P N P A V D A L A T D Q P A K P S P K K R K L S P E
E K Q A R E D A K K A K E E E R K L Q A K Q R E D A K N K K E E E K R L L A L A R E E E K K K R D E E K R V K D Q A K E E A R L K K E A E K K A R D D
A K L Q K D L E K K A K E E A R L Q K L Q E K Q K Q E E E E K R K K E R Q Q A V L A N F I K V A P V E A P R K P Q K V D P D L L I Q P F V C G S E F V
F P P S Y L R T R Q P S P G F D D A I A H K D G Q I T I E K L Q V E F R E H M H G K R N R K R D K K R R A E K L Q G L P P A L S N M K L L R F H D N
V R P A Y Y G T F S K R S A V L T G R R P L R T D D A V F D Y E V D S D A E W E D E G E G E E L S E S E G E K D K E E E E S E E E E E D K W V V P D
G Y L S E D E G M D S D D E K S K R A M K L K A E E G G E G G D A D T A S S S S G K R P A E H M E R D K T K R E K R E K K P C I I S T T F D P D S L
D S S A K A I L T N L S V Q P L V D C P I D L D W K P P A D P E E Q S A D S R S S K Q V V P D S L M P A L I L Y I H T H P M S L H K I I E G F H S E N T
Q V S K R Q L E L K I R E I A V K V K K S S W Q V R E E V V K S Y D V T L P E I I E S K P V P P K T P Q K N M T D F F A K A A G A K N Q P P N S N S N
S N S N S N P N S N S S T P T K K T P T K N T P T K N L P T P S S H T L P S E N S T K N A P T E N G C H T T P K R N I M D M F Q K H Q K T Q G

HIRA

M P A Q G I K C N P S Q P R A C A K F E D Q Q N T S Y N E Q M R I L K P D W V T H T G Y P I F S V D I H P D G S R F A T G G G D N K V I I W A S A
P V Q Y E E Q E A N N S C P R K L A E L T Y H M S P I S C V R W S H N G Q F L A S G S D D A L V M L W K L S S G P A S K S F G S D E L T Q E N W R
C T A T L R G H S K D I T D V A W S P D D S Q L A S C S V D N E I L V W D T A K N S V V A K L S G H T G F V K G V A W D P M G R Y I A S Q S E D K
T M I V W R C S D W K I E T K I E E P F V R S A A T T F Y R R P S W S P D G Q F I S S A H A F S G S N H V A M I T A R D T W R S V C D F V G H K L P
I V V T R F N P V I F A D T A G N N Y G V C L I G G Q D N T I S V W T T I S S R P L L A L N S C F K Q S V L D A S W A K D G F S F M C C S T D G T V V
W V E F A K G E L G T A L S P K E M D K V L R S I Y G D H I L S R P K S T V T V A E D P S Q L Y L P K T T A P M K P V A A Q P P P V P T Q Q V E T R L
A N G K R R I Q P Q F L G N I A S T T A T T P F I P P P T P F S S S S S S S T P F P S S S S S S S S F P A P S A F S S S S L P T P L P T A A Q V N G L Q V P A
N S L S S N G L I T S S S V N S S V M S S A T T N H S T T S S A V P V F I P P T P L P G S K S T S P V A V R H T I S L P D V G K R K E S D D V V E V P P P
T K K H K K D E E S D K E K G K E A E K G D K N K D I E A D H T K E K E R E K E R D K D R E K E R R R R E K E K E K E K V R K E K E K E R E A T K E S T
G G T K E R H M L S R L H I A H A Q H A P K L C K L I W E P T G L Q L E T K T E G M G T Q V S C R N T K F P Q Q L W R S S I P G K A V L V C G N Q
S F S A V A M E D S L L Y I F S A V G R L I F P P I S M S A P L S F L E A N K N F Y L A A I T S D C L L H V W N I A Q Q C A T I T A Q P V N I S P P S N L
H I S H L V L S D N G Q P V I I C S S H T Y A F H T E M K V W M K I D S V S S P S T G I T D G F L S K L M A T T E S G N R N I A T M T Q E S D E M L G
G L L S M E S H L A A S L V V G S S V E Y K Y W L L T Y T R Y L S S E A D V N R L T E L C K S L L G P P T G K T G P A P T P A Q G K N W E P S I L G M
A K R E L L R E L L P I M S T N R S L Q R L V A Q F R E G L E S I S K

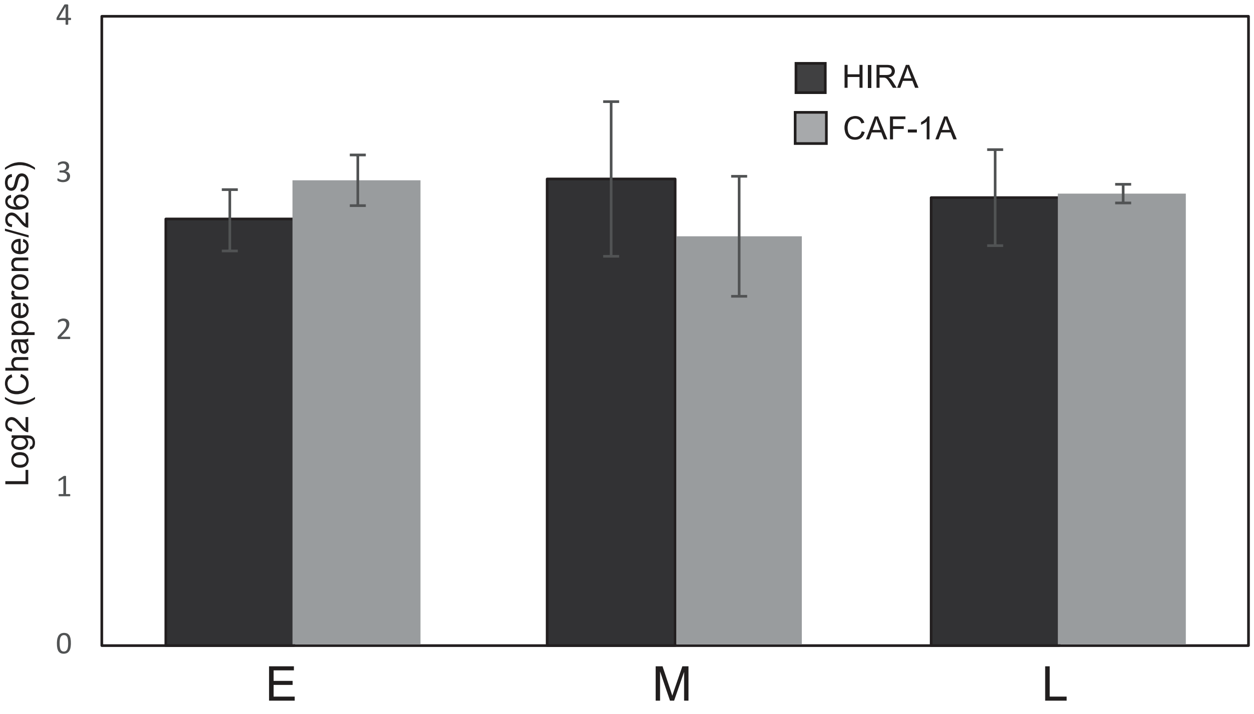

Fig.S7 Expression of the chaperones CAF-1A and HIRA during S-phase: CAF-1A corresponds to the sequence of the largest sub-unit of CAF-1 in Physarum. HIRA corresponds to the sequence of the Physarum HIRA. The bar graph represents the expression of HIRA/CAF-1A determined by qRT-PCR in early (E), mid (M) and late (L) S-phase. The quantifications were performed relative to 26S rRNA.
