# Supplementary material for: Usage of the H3 variants during the S-phase of the cell cycle in Physarum polycephalum
Source: Nucleic Acids Res. 2022 Feb 7;50(5):2536–48. doi: 10.1093/nar/gkac060 (PMC8934661; doi:10.1093/nar/gkac060)
Supplement: gkac060_Supplemental_Files [file gkac060_supplemental_files.zip › list of primers & supp figures.pdf]

Name primer	Sequence 5' → 3'
Pp1 US	ACG GAG ATC AGT CTT GAA GTC CTG
Pp1 DS	TTC TTG ATT CCA CCA GAG GTG GGT
Pp2 US	GTC CTG ATG TTC TGA CTG AAC GCA
Pp2 DS	CAT AAG CGC CTC TTG CAC GCA TTT
Pp1 US Q	TTT ACG CAC GTT CTC CGC GAA TAC
Pp1 DS Q	AGG CCT ACT TGG TTG GTC TCT TTG
Pp2 US Q	TCC GTC GTT ACC AAA AGA GCA CTG
Pp2 DS Q	AAA GAG ACC AAC CAA GTA GGC CTC
Pp 26S US	CGA AAT CCA ACC AAG CTC GGG TAA
Pp 26S DS	CTT GGC ACA ATT AGC GGG GAA AGA
Pp CAF-1A US	CAA AAC CAT TAC CTC CAG ACC TCC
Pp CAF-1A DS	GGT TGA TCG GTT GCT AAA GCA TCC
Pp HIRA US	TTT CAC TGG CAG AAC GAG CAA TGG
Pp HIRA DS	TAT TGG GTA TCC TGT GTG GGT GAC

Name siRNA	Sequence 5' → 3'
Pp1H33a	UUAGUUCUUCGGAGUGUCCAAGUCC
Pp1H33b	UUCUGACUGAACGCAAAGGUUCGGA
Pp2H3a	AACGUUUGGUGCGUGAGAUUGCUCA
Pp2H3b	AAGUUGCCCUUCCAACGUUUGGUGC
Control	AGGUAGUGUAAUCGCCUUG

Table S1 List of the primers and siRNAs
